# Mechanism of emodin in treating hepatitis B virus-associated hepatocellular carcinoma: network pharmacology and cell experiments

**DOI:** 10.3389/fcimb.2024.1458913

**Published:** 2024-09-13

**Authors:** Yupeng Wang, Shuangxing Li, Tianqi Ren, Yikun Zhang, Bo Li, Xingchao Geng

**Affiliations:** ^1^ National Center for Safety Evaluation of Drugs, National Institutes for Food and Drug Control, Chinese Academy of Medical Sciences and Peking Union Medical College, Beijing, China; ^2^ National Center for Safety Evaluation of Drugs, National Institutes for Food and Drug Control, Beijing, China; ^3^ School of Basic Medicine and Clinical Pharmacy, China Pharmaceutical University, Nanjing, China

**Keywords:** emodin, hepatitis B virus-related hepatocellular carcinoma, network pharmacology, a systematic study, molecular docking validation

## Abstract

**Introduction:**

Hepatocellular carcinoma (HCC) is a pressing global issue, with Hepatitis B virus (HBV) infection remaining the primary. Emodin, an anthraquinone compound extracted from the natural plant’s. This study investigates the molecular targets and possible mechanisms of emodin in treating HBV-related HCC based on network pharmacology and molecular docking and validates the screened molecular targets through *in vitro* experiments.

**Methods:**

Potential targets related to emodin were obtained through PubChem, CTD, PharmMapper, SuperPred, and TargetNet databases. Potential disease targets for HBV and HCC were identified using the DisGeNET, GeneCards, OMIM, and TTD databases. A Venn diagram was used to determine overlapping genes between the drug and the diseases. Enrichment analysis of these genes was performed using GO and KEGG via bioinformatics websites. The overlapping genes were imported into STRING to construct a protein-protein interaction network. Cytoscape 3.9.1 software was used for visualizing and analyzing the core targets. Molecular docking analysis of the drug and core targets was performed using Schrodinger. The regulatory effects of emodin on these core targets were validate through *in vitro* experiments.

**Results:**

A total of 43 overlapping genes were identified. GO analysis recognized 926 entries, and KEGG analysis identified 135 entries. The main pathways involved in the KEGG analysis included cancer, human cytomegalovirus infection and prostate cancer. The binding energies of emodin with HSP90AA1, PTGS2, GSTP1, SOD2, MAPK3, and PCNA were all less than -5 kcal/mol. Compared to normal liver tissue, the mRNA levels of XRCC1, MAPK3, and PCNA were significantly elevated in liver cancer tissue. The expression levels of XRCC1, HIF1A, MAPK3, and PCNA genes were closely related to HCC progression. High expressions of HSP90AA1, TGFB1, HIF1A, MAPK3, and PCNA were all closely associated with poor prognosis in HCC. *In vitro* experiments demonstrated that emodin significantly downregulated the expression of HSP90AA1, MAPK3, XRCC1, PCNA, and SOD2, while significantly upregulating the expression of PTGS2 and GSTP1.

**Conclusion:**

This study, based on network pharmacology and molecular docking validation, suggests that emodin may exert therapeutic effects on HBV-related HCC by downregulating the expression of XRCC1, MAPK3, PCNA, HSP90AA1, and SOD2, and upregulating the expression of PTGS2 and GSTP1.

## 1 Introduction

Hepatocellular carcinoma (HCC) is a common malignant tumor with widespread global impact, particularly in developing countries where both incidence and mortality rates are high. According to statistics from the World Health Organization, HCC is one of the leading causes of cancer-related deaths (Wang and Deng, 2023). Hepatitis B virus (HBV) infection is the most common factor leading to HCC worldwide, particularly in Asia (de Martel et al., 2015). Current data indicate that HBV infection is positively correlated with the risk of developing HCC (Rizzo et al., 2022). This association makes the prevention and treatment of HBV a crucial strategy in controlling HCC incidence. Presently, almost all HCC management guidelines recommend routine antiviral therapy to prevent HBV reactivation during HCC treatment, thereby reducing the recurrence of HCC after curative treatment (Sarin et al., 2016; Terrault et al., 2018; Di Dato and Iorio, 2024). Antiviral therapy not only controls HBV replication and reduces liver inflammation and fibrosis but also lowers the risk of HCC development. In HCC patients, antiviral therapy has been shown to significantly improve prognosis, reducing HCC recurrence rates and mortality. However, despite the availability of various antiviral drugs that effectively inhibit HBV replication, no drugs that simultaneously suppress both HBV infection and HCC have yet been marketed. Although existing anti-HBV drugs can prevent HCC to some extent, their therapeutic efficacy is limited for those who have already developed HCC. The treatment of HCC remains a significant challenge, especially in patients with advanced and recurrent HCC. Therefore, there is an urgent need to develop drugs with both anti-HBV and anti-HCC activities. Such dual-action drugs could effectively control HBV infection, reduce the risk of HCC development, and directly inhibit the growth and spread of HCC, thereby significantly improving patient survival rates and quality of life.

In recent years, an increasing number of studies have revealed that traditional Chinese medicine (TCM) has unique advantages in treating liver diseases and has identified many potentially valuable small molecule compounds (Khan et al., 2019; Izzo et al., 2021). These compounds not only exhibit diverse biological activities but also act on multiple targets and pathways, highlighting the advantages and potential of TCM in the treatment of complex diseases. Emodin (1,3,8-trihydroxy-6-methyl-anthraquinone) is an active ingredient extracted from TCM herbs such as Polygonum multiflorum, Rheum palmatum, and Cassia obtusifolia, and has been widely studied in recently years for its various pharmacological effects. Specifically, emodin not only possesses anti-HBV activity but also exhibits antioxidant, hepatoprotective, and antitumor properties (Dong et al., 2016; Tuli et al., 2021; Shao et al., 2022). Studies have shown that emodin can effectively inhibits HBV DNA replication and the secretion of hepatitis B surface antigen (HBsAg) (Shuangsuo et al., 2006). This is of great significance for controlling HBV infection and reducing virus transmission. Additionally, emodin has also demonstrated significant antitumor effects. Research has found that emodin can induce apoptosis in hepatocellular carcinoma cells through multiple pathways, thereby inhibiting the occurrence of hepatocellular carcinoma, with specific mechanisms involving the death receptor pathway and mitochondrial apoptosis pathway (Hu et al., 2020). Therefore, emodin has significant advantages in treating HBV-related HCC (HBV-HCC). Its multiple pharmacological actions not only directly inhibit HBV infection but also suppress the occurrence and progression of hepatocellular carcinoma through various mechanisms. However, despite the preliminary confirmation of emodin’s therapeutic effects, its specific targets and signaling pathways require further research and exploration.

With the rapid development of bioinformatics, network pharmacology based on large databases has become a powerful tool for characterizing the mechanisms of complex drug systems from the cellular level to the molecular level (Zhou et al., 2020). Network pharmacology, based on systems biology, multi-directional pharmacology, and high-throughput analysis, can thoroughly explain the complex relationships between drugs and diseases by constructing bio-network visualizations of potential active ingredients, hub targets, signaling pathways, and diseases. Many studies have used network pharmacology methods to reveal the mechanisms of drug actions on diseases (Fan et al., 2022; Shang et al., 2023; Xu et al., 2024). Molecular docking is a computer simulation technique that models the interactions between molecules and proteins at the atomic level, predicts the conformations of ligands and receptors, and calculates parameters such as affinity to evaluate the binding situation between molecules and proteins (Paggi et al., 2024). This technique is relatively accurate and cost-effective, and it is currently mainly used in drug design and elucidation of biochemical pathways. Therefore, this study uses network pharmacology and molecular docking techniques to explore the targets and possible molecular mechanisms of emodin in the treatment of HBV-HCC.

## 2 Materials and methods

### 2.1 Network pharmacology

#### 2.1.1 Target prediction for emodin

**Figure 1:**
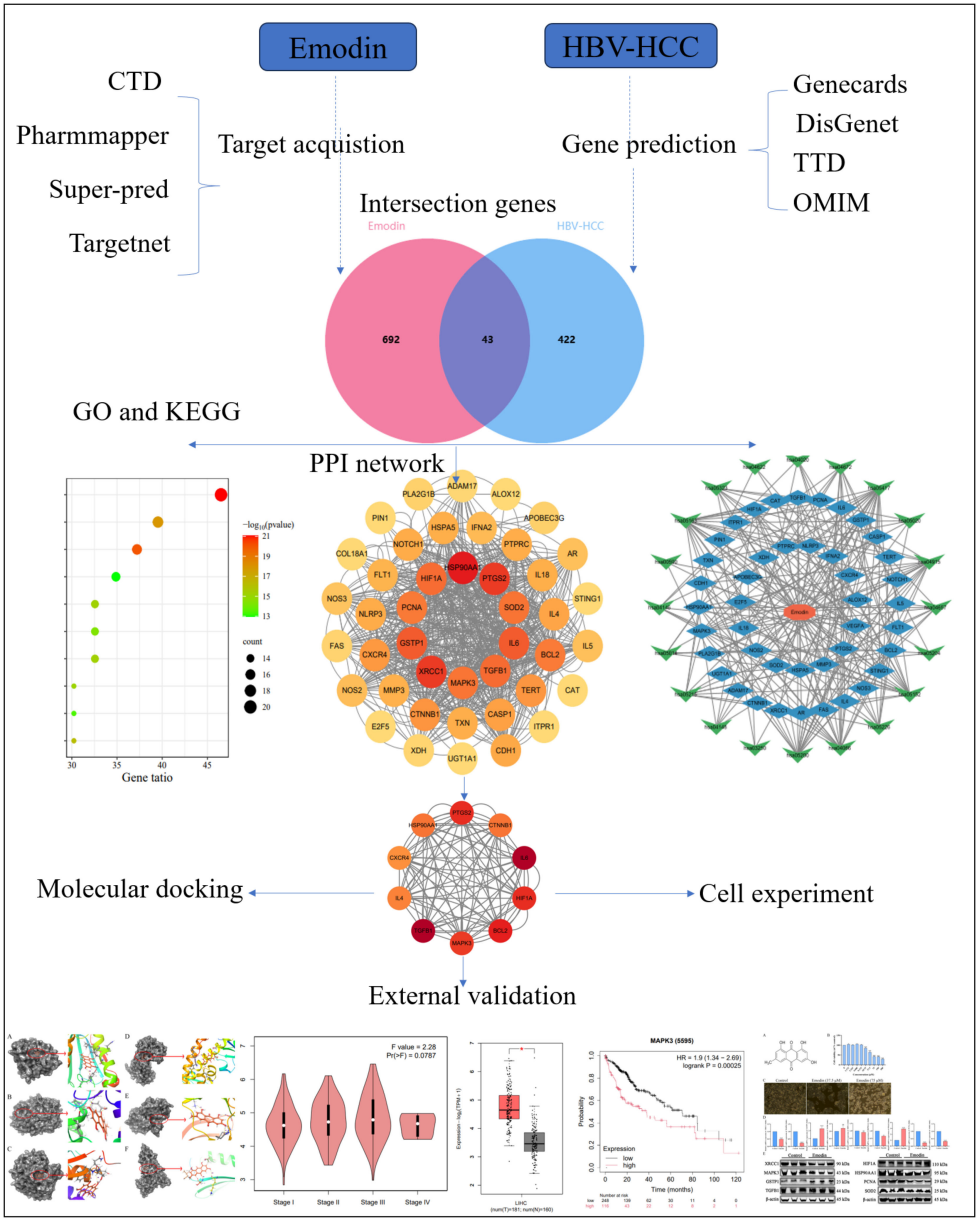
Detailed graphical summary of network pharmacology (including the network pharmacology process, molecular docking, external validation of core targets and cell experiment).

The workflow of this analysis is illustrated in **Figure 1**. The SMILES of emodin was obtained from the PubChem database to acquire its chemical structure, chemical properties, and biological activities. Potential target genes were identified by screening the Comparative Toxicogenomics, PharmMapper, SuperPred, and TargetNet databases. After removing duplicates, the selected targets were standardized using the UniProt database. The genes screened from these four databases under specific conditions are those associated with the therapeutic effects of emodin (Nickel et al., 2014; Yao et al., 2016; Wang et al., 2017; Davis et al., 2023). Emodin may exert its therapeutic effects through the interactions among these genes. The PPI data were obtained from the STRING database. Human species were set as a requirement for this analysis. Topological parameters were calculated using the CytoNCA plugin of Cytoscape.

#### 2.1.2 Identification of potential targets in HBV-HCC and intersection genes

This study utilized the DisGeNET, GeneCards, OMIM, and TTD databases to identify genes associated with HBV-HCC (Amberger et al., 2015; Stelzer et al., 2016; Piñero et al., 2021; Zhou et al., 2022). Based on rankings, we screened for mutation genes more likely to be involved in the occurrence and progression of HBV-HCC and normalized these disease-related genes using UniProt. Subsequently, the drug and disease genes were located, and the location results were imported into the jevnn website to obtain intersecting genes (Bardou et al., 2014). The therapeutic effect of emodin on HBV-HCC may be mediated by regulating these intersecting genes.

#### 2.1.3 GO and KEGG enrichment analysis

GO is a biological system used to study the impact of individual genes on organisms at different biological levels. KEGG provides evidence of the involvement of individual genes in biological signaling pathways (Kanehisa et al., 2022). In this study, GO and KEGG enrichment analysis was conducted using the GO and KEGG online analysis modules available on the Metascape website. After importing the list of intersecting genes and selecting “Homo sapiens” as the species, the program was run until the enrichment analysis results and related images were exported.

#### 2.1.4 Drug-target-pathway network construction

By importing the intersecting genes and KEGG signaling pathway entries into Cytoscape 3.9.1, a drug-target-pathway network was established. Cytoscape is a platform that integrates complex network structures and data, presenting this information in graphical form (Shannon et al., 2003). In the network, nodes represent emodin, intersecting genes, or pathways, while the edges between nodes indicate the involvement of genes in different pathways.

#### 2.1.5 Protein-protein interaction network construction and visualization

PPI is one method to study the mechanisms by which proteins function within cells (Ding and Kihara, 2019). A PPI network was constructed using the STRING database, with the species limited to “Homo sapiens” and a medium confidence score set at 0.4 to ensure the inclusion of more protein-protein interaction information. After removing disconnected nodes, the PPI network was updated. Subsequently, a tab-separated values (tsv) file was downloaded and imported into Cytoscape 3.9.1 for further visualization. The interaction strengths between proteins were calculated in Cytoscape to screen for core targets.

#### 2.1.6 Screening of core targets

Core targets were determined based on Betweenness values using the “CytoNCA” plugin in Cytoscape 3.9.1. “CytoNCA” is a plugin used to calculate the strength of protein interactions, allowing for the identification of proteins with higher interaction strength with other proteins as core targets. The plugin includes various methods for calculating protein interaction strength, one of which was selected for screening core targets. Intersecting genes with betweenness values more than twice the median were chosen as core targets.

#### 2.1.7 Molecular docking between emodin and core targets

To further elucidate the interaction between the candidate proteins and emodin, as well as their mechanisms of action, we conducted molecular docking to determine the interaction strength between receptors and ligands. We downloaded the SDF format file of emodin from PubChem and obtained the SDF format file of the original ligand from the Protein Data Bank (PDB). Additionally, the PDB format files of the receptor proteins were also acquired from the PDB database (**Table 1**). We imported the SDF files of emodin and the original ligand, along with the PDB files of the receptor proteins, into Schrodinger software. First, we performed dehydration and degreasing on the proteins, followed by hydrogenation. Next, we used the Protein Preparation Wizard module in Schrodinger to preprocess the proteins and employed the LigPrep module to preprocess the ligands, including emodin and the original ligand, before docking. To better simulate the molecular docking situation, we used the Induced Fit Docking (IFD) module in Schrodinger for induced fit docking. We selected “Box center: Centroid of Workspace Ligand,” generated appropriate docking boxes, and recorded the corresponding parameters (**Table 2**). We then set the appropriate docking parameters and performed the induced fit docking (IFD) to generate result files in Compressed Maestro File format. These files were then re-imported into Schrodinger for visualization of the molecular docking results, and the docking results data were recorded in the workstation table.

**Table 1:** Details of the protein targets in the PDB database.

Targets	PDB ID	Method	Resolution	R-value free	R-value work	R-Value observed
HSP90AA1	3O0I	X-RAY DIFFRACTION	1.47 Å	0.234	0.207	0.209
PTGS2	5KIR	X-RAY DIFFRACTION	2.70 Å	0.220	0.178	0.180
GSTP1	6LLX	X-RAY DIFFRACTION	1.58 Å	0.223	0.200	0.201
SOD2	7KKU	X-RAY DIFFRACTION	2.02 Å	0.252	0.217	0.219
MAPK3	6GES	X-RAY DIFFRACTION	2.07 Å	0.213	0.170	0.172
PCNA	3WGW	X-RAY DIFFRACTION	2.80 Å	0.221	0.182	0.184

**Table 2:** Induced Fit Docking parameters in molecular docking.

Targets	PDB ID	Box center: Centroid of Workspace Ligand
HSP90AA1	3O0I	B:601
PTGS2	5KIR	B:302
GSTP1	6LLX	B:401
SOD2	7KKU	A:303
MAPK3	6GES	A:207
PCNA	3WGW	A:202

#### 2.1.8 External validation of core targets

We analyzed the transcript and protein levels of 10 core targets in HCC cells and normal liver cells using the Gene Expression Profiling Interactive Analysis (GEPIA) database and the Human Protein Atlas (Uhlén et al., 2015; Tang et al., 2017). Pathological staging analysis of core targets in HBV-HCC cells was conducted to verify changes in mRNA expression at different stages of the disease. Subsequently, we investigated the impact of core target mutations on the prognosis of HCC patients and analyzed the effect of core target expression differences on the survival rates of HCC patients using the Kaplan-Meier Plotter database (Győrffy, 2023). All websites used in this analysis are shown in **Table 3**.

**Table 3:** Basic information of the database used for the screening of emodin in the treatment of HCC.

Database	Website
Pubchem	https://pubchem.ncbi.nlm.nih.gov
CTD	https://ctdbase.org
PharmMapper	http://lilab-ecust.cn/pharmmapper/index.html
SuperPred	https://prediction.charite.de
TargetNet	http://targetnet.scbdd.com/
Uniprot	https://www.uniprot.org/
DisGeNET	https://www.disgenet.org/
GeneCards	https://www.genecards.org/
OMIM	https://www.omim.org/
TTD	https://db.idrblab.net/ttd/
Jvenn	https://jvenn.toulouse.inra.fr/app/index.html
STRING	https://cn.string-db.org/
PDB	https://www.rcsb.org/
GEPIA	http://gepia.cancer-pku.cn/
HPA	https://www.proteinatlas.org/
Kaplan-Meier plotter	https://kmplot.com/
Metascape	https://metascape.org/

### 2.2 Biological testing

#### 2.2.1 Test compound and cell culture

Emodin standard was provided by the National Institutes for Food and Drug Control of China, dissolved in dimethyl sulfoxide (8.1 mg: 1 ml), and stored at -20°C. The concentration of Emodin in different experiments was achieved by diluting with DMEM. HepG2 cells were maintained in DMEM supplemented with 10% fetal bovine serum (Gibco), and 1% penicillin and streptomycin (100 U/ml).

#### 2.2.2 Cell viability assay

HepG2 cells were seeded into a 96-well plate at a density of 2×10^4^ cells per well and then incubated with Emodin solutions of varying concentrations for 24 hours. Subsequently, 10 μl of CCK-8 solution (#CK04, DOJINDO, Japan) was added to each well, and the plate was incubated for an additional hour in the incubator. The optical density (OD) was measured at 450 nm. Cell viability was calculated using the formula: Cell viability = (OD of the experimental group - OD of the blank group)/(OD of the control group - OD of the blank group) × 100%.

#### 2.2.3 mRNA expression of core targets

HepG2 cells were seeded into six-well plates (1×10^6^ cells/well) and cultured overnight. After attachment, the cells were exposed to emodin for 24 hours. Total RNA was isolated from the cells using the FastPure Cell/Tissue Total RNA Isolation Kit (#RC112-01, Vazyme Biotech Co., Ltd), and the RNA purity and concentration were measured using a NanoDrop-1000 spectrophotometer. Subsequently, first-strand cDNA synthesis was performed using the HiScript III RT SuperMix (#R323-01, Vazyme Biotech Co., Ltd). RT-qPCR was conducted using Taq Pro Universal SYBR qPCR Master Mix (#Q717-02, Vazyme Biotech Co., Ltd). β-actin was used as the internal reference to measure the transcription levels of the target genes. Relative gene transcription was calculated using the 2 ^−ΔΔCT^ method, and the data were expressed as fold differences relative to the control group. All primer sequences used in this study are listed in **Table 4**.

**Table 4:** Basic information of primer sequences.

Primer Name	Sequence (5’ to 3’)
Hsp90AA1-F	TCTGCCTCTGGTGATGAGATGG
Hsp90AA1-R	CGTTCCACAAAGGCTGAGTTAGC
PGST2-F	CGGTGAAACTCTGGCTAGACAG
PGST2-R	GCAAACCGTAGATGCTCAGGGA
GSTP1-F	TGGACATGGTGAATGACGGCGT
GSTP1-R	GGTCTCAAAAGGCTTCAGTTGCC
SOD2-F	CTGGACAAACCTCAGCCCTAAC
SOD2-R	AACCTGAGCCTTGGACACCAAC
MAPK3-F	TGGCAAGCACTACCTGGATCAG
MAPK3-R	GCAGAGACTGTAGGTAGTTTCGG
PCNA-F	CAAGTAATGTCGATAAAGAGGAGG
PCNA-R	GTGTCACCGTTGAAGAGAGTGG
XRCC1-F	CGGATGAGAACACGGACAGTGA
XRCC1-R	GAAGGCTGTGACGTATCGGATG
TGFB1-F	TACCTGAACCCGTGTTGCTCTC
TGFB1-R	GTTGCTGAGGTATCGCCAGGAA
HIF1A-F	TATGAGCCAGAAGAACTTTTAGGC
HIF1A-R	CACCTCTTTTGGCAAGCATCCTG
β-ACTIN-F	CACCATTGGCAATGAGCGGTTC
β-ACTIN-R	AGGTCTTTGCGGATGTCCACGT

#### 2.2.4 Western blot analysis of core targets

HepG2 cells were seeded into six-well plates (1×10^6^ cells/well) and cultured overnight. After attachment, the cells were exposed to emodin for 24 hours. The treated cells were lysed using RIPA buffer containing 1% protease and phosphatase inhibitors, and total proteins were extracted using a total protein extraction kit. Thirty micrograms of protein were separated by 10% sodium dodecyl sulfate-polyacrylamide gel electrophoresis (SDS-PAGE) and then transferred onto a PVDF membrane. After blocking the membrane with 5% milk for 1 hour, it was incubated overnight at 4°C with the primary antibody. Following three washes with TBST, the membrane was incubated at room temperature with horseradish peroxidase (HRP)-conjugated secondary antibody for 1 hour. After incubation, the PVDF membrane was washed three times, and the ECL chemiluminescence signals of the protein bands were captured and imaged using the iBright imaging system.

## 3 Results

### 3.1 Network pharmacology based-analysis

#### 3.1.1 Identification of targets and intersection genes

**Figure 2:**
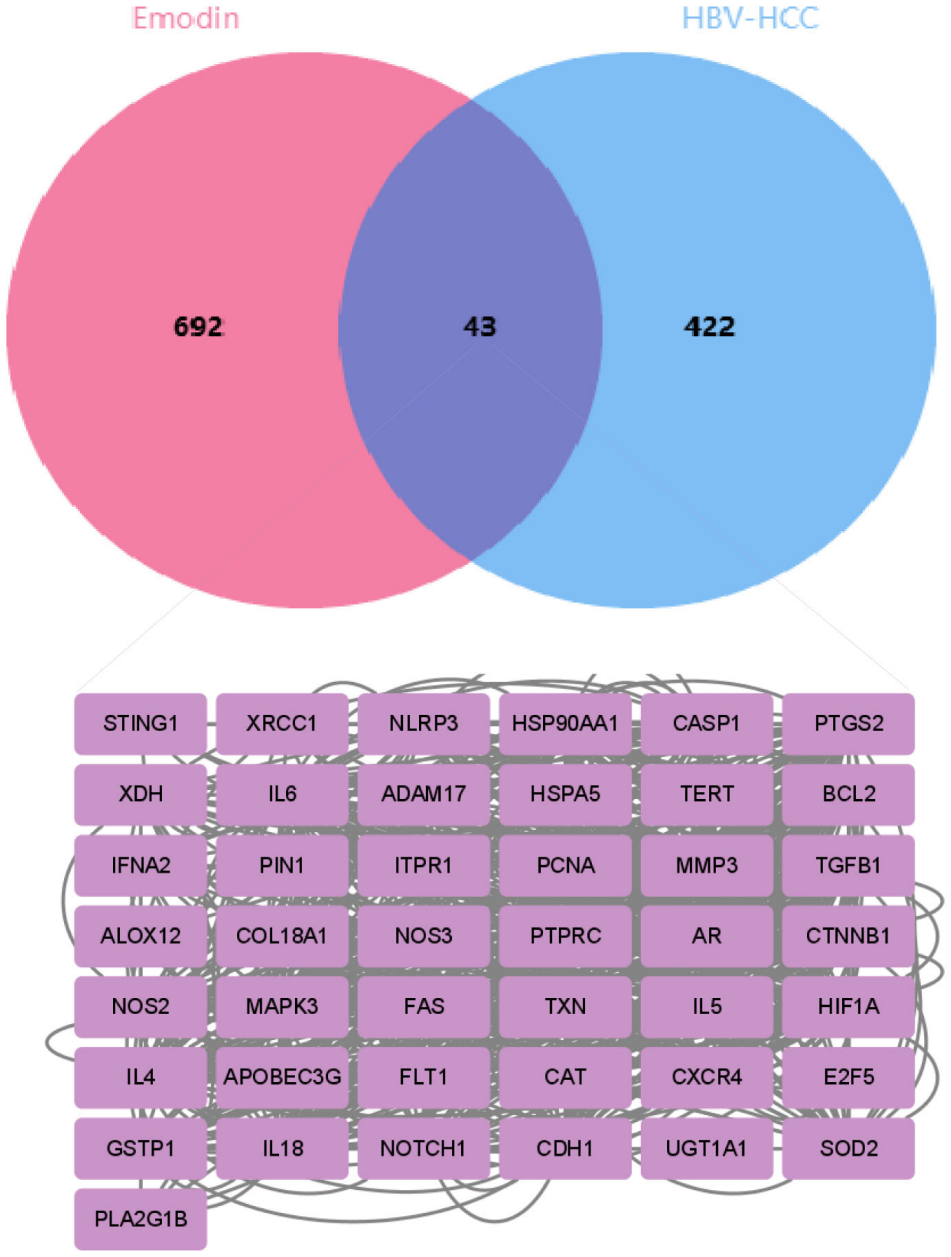
The Venn diagram shows the overlapping genes between emodin and HBV-HCC. The pink section represents 735 emodin-related genes screened from multiple databases, the blue section represents 465 HBV-HCC-related genes screened from the database, and the intersection in the middle represents 43 overlapping genes.

Based on a normalized fit score of ≥0.6, 50 genes were identified from the PharmMapper database. Additionally, 284 and 159 genes were screened from the SuperPred and TargetNet databases, respectively, based on a probability of ≥0.5. From the CTD database, 245 genes were selected. After removing duplicates, a total of 735 drug-related genes were obtained. Concurrently, 465 disease-related genes were identified from the DisGeNet, GeneCards, OMIM, and TTD databases. Through Venn diagram analysis (**Figure 2**), 43 intersecting genes were matched. These intersecting genes may play significant regulatory roles in the occurrence and development of HBV-HCC. To further understand the cancer pathways enriched by these intersecting genes, we conducted GO and KEGG enrichment analyses to reveal the regulatory mechanisms of emodin on HBV-HCC.

#### 3.1.2 GO and KEGG enrichment analysis

**Figure 3:**
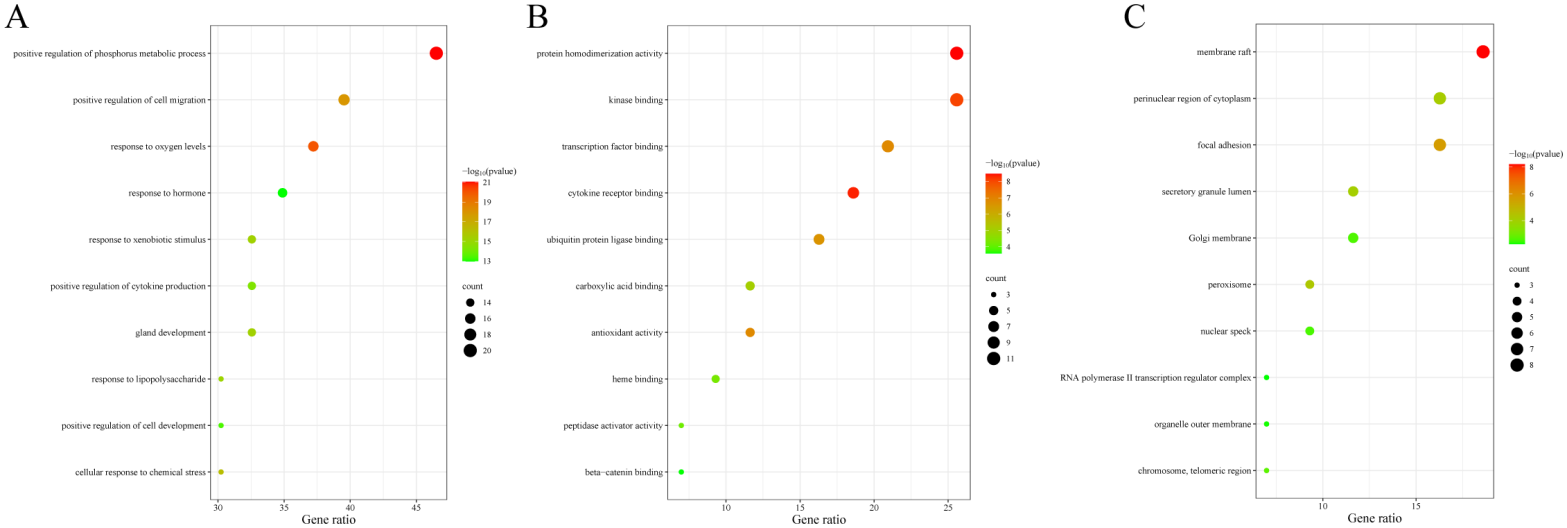
Bubble chart of GO functional analysis of emodin in HBV-HCC. The color of the bubbles indicates the p-value, and the size of the bubbles represents the number of genes involved in the biological process. **(A)** Biological processes of emodin in HBV-HCC. **(B)** Cellular components of emodin in HBV-HCC. **(C)** Molecular functions of emodin in HBV-HCC.

**Figure 4:**
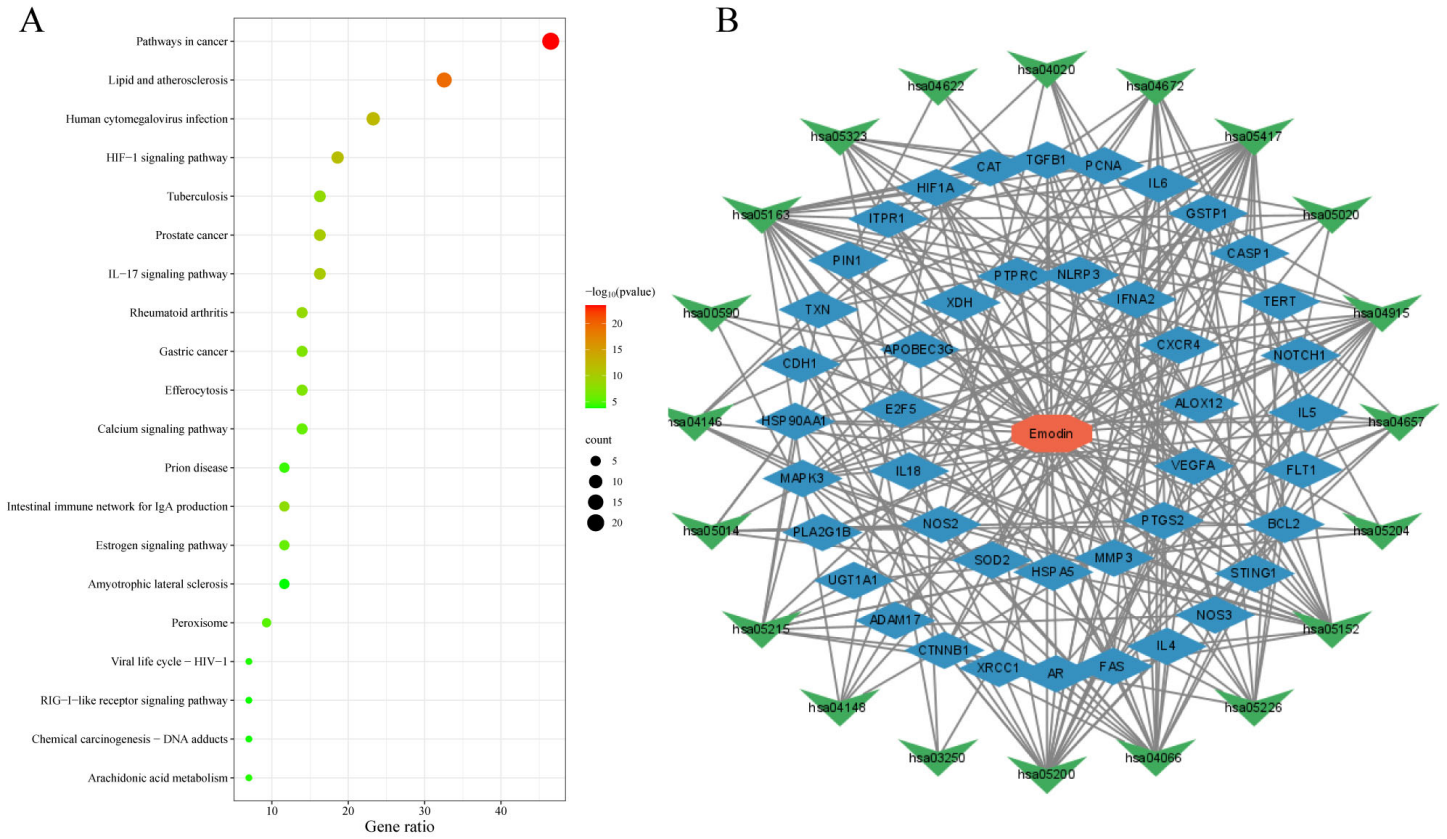
Bubble chart of KEGG enrichment analysis of emodin in HBV-HCC. **(A)** KEGG pathway enrichment analysis of emodin in HBV-HCC. The red color of the bubbles indicates smaller p-values, suggesting that these intersecting genes are more likely involved in regulatory pathways. The size of the bubbles represents the number of genes involved in regulating the pathway. **(B)** Drug-target-pathway network diagram. The red octagon represents emodin, the blue diamond represents targets, and the green triangles represent pathways. This diagram indicates that emodin may exert its therapeutic effects on HBV-HCC by regulating multiple targets and pathways.

The data were input into bioinformatics websites for further GO and KEGG enrichment analyses. GO analysis generated 926 entries, categorized into biological processes (n=823), cellular components (n=42), and molecular functions (n=61). The top 10 entries of each type were filtered based on *p-*value (**Figure 3**). The biological process ontology primarily included positive regulation of cytokine production, positive regulation of cell development, positive regulation of cell migration, and response to hormones. The cellular component ontology consisted of membrane raft, membrane microdomain, membrane region, peroxisome, chromosome, and transcription regulator complex. The molecular function ontology included cytokine receptor binding, kinase binding, transcription factor binding, and ubiquitin protein ligase binding. KEGG enrichment analysis identified 135 entries, with the top 20 KEGG signaling pathways filtered based on *p*-value (**Figure 4A**). The main enriched pathways included cancer pathways, human cytomegalovirus infection pathways, tuberculosis pathways, intestinal immune network for IgA production pathways, gastric cancer pathways, prion disease pathways, and prostate cancer pathways. Visualization of genes involved in each signaling pathway using Cytoscape 3.9.1 indicated that the activation of multiple signaling pathways might play a role in the therapeutic effect of emodin on HBV-HCC (**Figure 4B**, **Table 5**). Therefore, emodin may exert its therapeutic effect on HBV-HCC by regulating these pathways enriched by the 43 intersecting genes. Emodin inhibits the proliferation of HBV-HCC cells by targeting one or more of these cancer pathways.

**Table 5:** Basic information of KEGG signal pathways.

Entry	Name
hsa04622	RIG-I-like receptor signaling pathway
hsa04020	Calcium signaling pathway
hsa04672	Intestinal immune network for IgA production
hsa05417	Lipid and atherosclerosis
hsa05020	Prion disease
hsa04915	Estrogen signaling pathway
Hsa04657	IL-17 signaling pathway
hsa05204	Chemical carcinogenesis - DNA adducts
hsa05152	Tuberculosis
hsa05226	Gastric cancer
hsa04066	HIF-1 signaling pathway
hsa05200	Pathways in cancer
hsa03250	Viral life cycle - HIV-1
hsa04148	Efferocytosis
hsa05215	Prostate cancer
hsa05014	Amyotrophic lateral sclerosis
hsa04146	Peroxisome
hsa00590	Arachidonic acid metabolism
hsa05163	Human cytomegalovirus infection
hsa05323	Rheumatoid arthritis

#### 3.1.3 PPI network construction and selection of core targets

**Figure 5:**
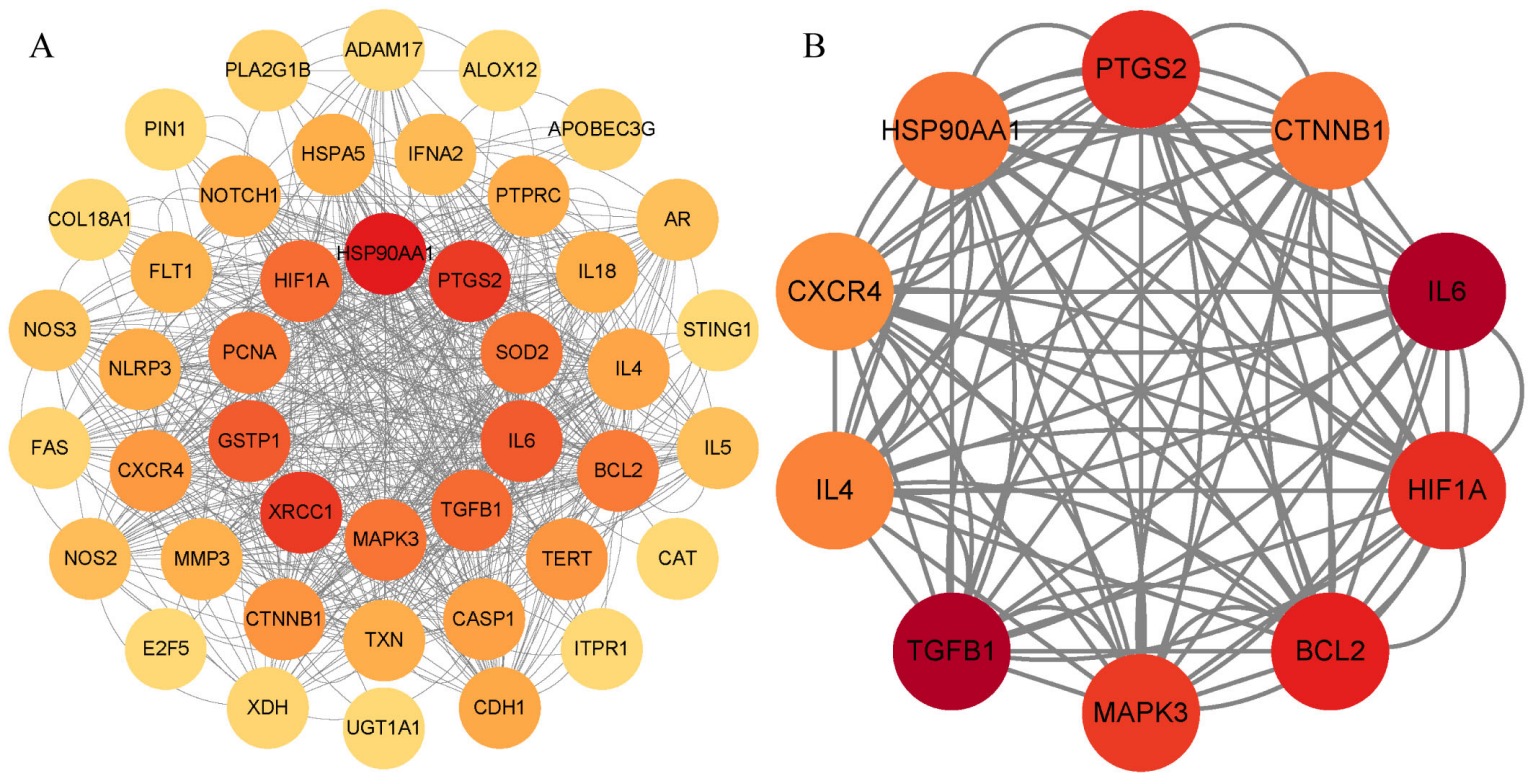
PPI network diagram. **(A)** PPI network of potential targets for emodin in the treatment of HBV-HCC. **(B)** Core targets are selected based on betweenness centrality values, with the color gradient representing the magnitude of the betweenness centrality values.

The PPI network comprised a total of 43 nodes and 308 edges, with an average node degree of 14.3 and a p-value of <1.0e-16, indicating that these proteins are at least biologically relevant. The PPI network was visualized using Cytoscape 3.9.1 (**Figure 5A**). We utilized the “Betweenness” algorithm within the “CytoNCA” plugin to calculate the betweenness centrality value of each protein. The Betweenness value represents the number of shortest paths passing through the node, with higher values indicating a more significant role of the protein in the interactions (**Table 6**). The intensity of the node color indicates the magnitude of its Betweenness value. The results showed that HSP90AA1, XRCC1, PTGS2, IL-6, GSTP1, TGFB1, HIF1A, SOD2, MAPK3, and PCNA might be key targets for emodin in the treatment of HBV-HCC (**Figure 5B**).

**Table 6:** 10 core gene identified using betweenness value by CytoNCA in Cytoscape.

Target name	Full name	Betweenness
HSP90AA1	Heat shock protein 90 alpha family class A member 1	201.2
XRCC1	X-ray repair cross complementing 1	160.6
PTGS2	Prostaglandin-endoperoxide synthase 2	160.4
IL-6	Interleukin 6	121.3
GSTP1	Glutathione S-transferase pi 1	121.1
TGFB1	Transforming growth factor beta 1	101.3
HIF1A	Hypoxia inducible factor 1 subunit alpha	99.8
SOD2	Superoxide dismutase 2	89.9
MAPK3	Mitogen-activated protein kinase 3	88.9
PCNA	Proliferating cell nuclear antigen	82

#### 3.1.4 Molecular docking validation of emodin and core targets

**Figure 6:**
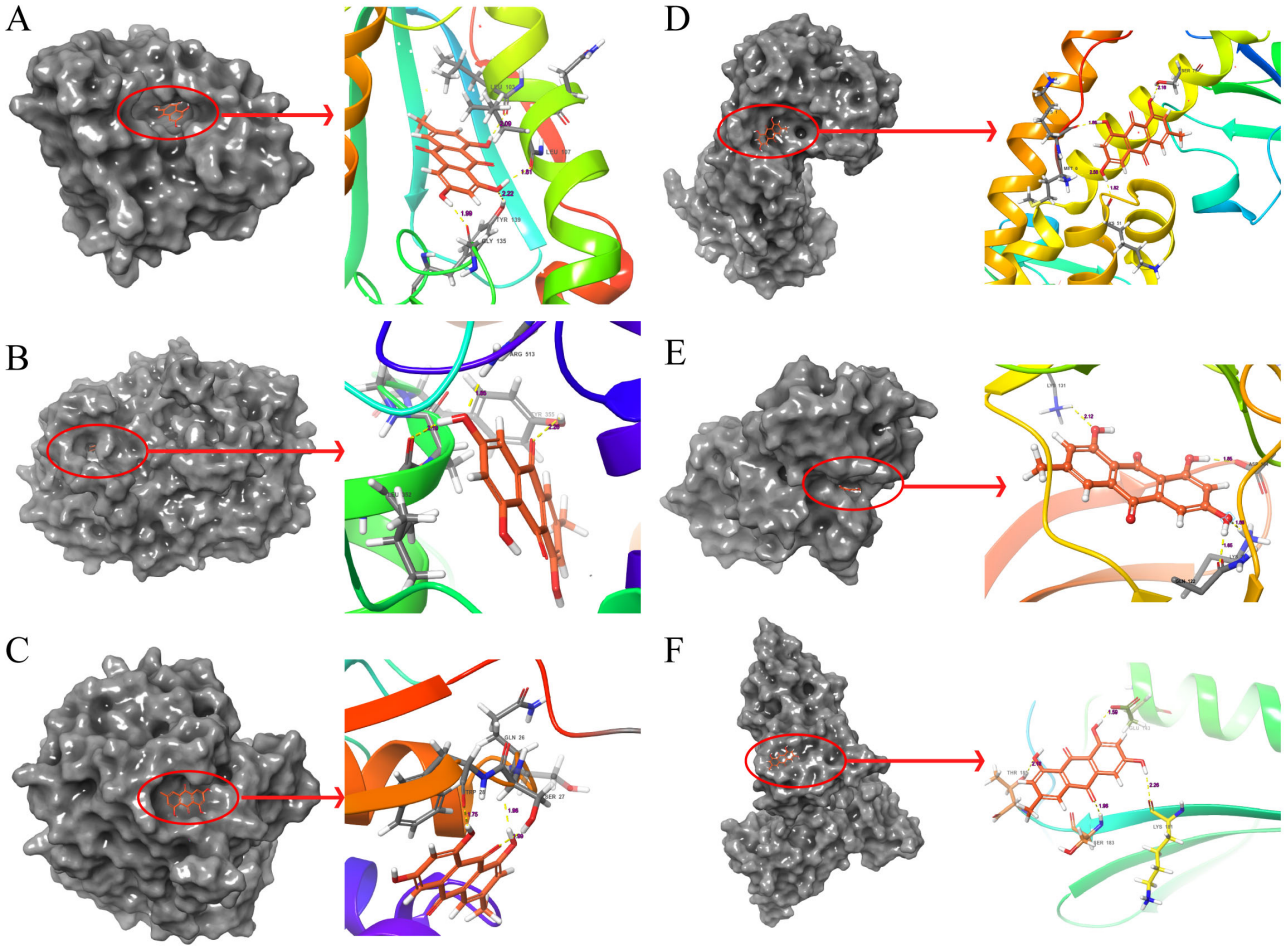
Molecular docking pattern of emodin and core target protein. The gray portion on the left represents the surface location of emodin on the protein receptor, and the right represents the name of the specific amino acid that emodin binds to each protein and the length and number of hydrogen bonds. **(A)** Emodin-HSP90AA1, **(B)** Emodin-PTGS2, **(C)** Emodin-GSTP1, **(D)** Emodin-SOD2, **(E)** Emodin-MAPK3, **(F)** Emodin-PCNA.

**Figure 7:**
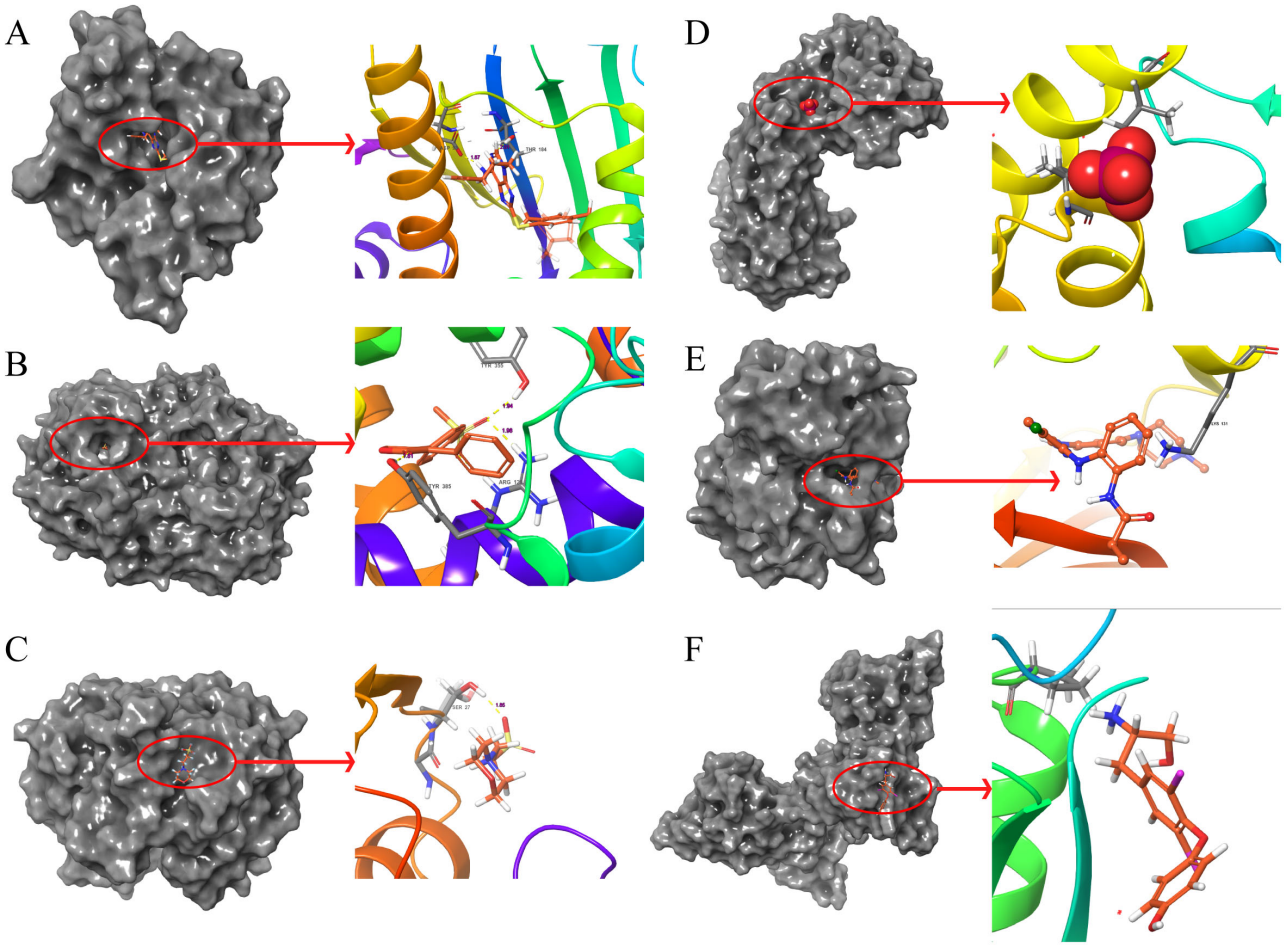
Molecular docking pattern of original ligand and core target protein. The gray portion on the left represents the surface location of their original ligands on the protein receptor, and the right represents the name of the specific amino acid that their original ligands bind to each protein and the length and number of hydrogen bonds. **(A)** 3O01-HSP90AA1, **(B)** 5KIR-PTGS2, **(C)** 6LLX-GSTP1, **(D)** 7KKU-SOD2, **(E)** 6GES-MAPK3, **(F)** 3WGW-PCNA.

The molecular docking results indicate that the docking energy values of emodin with HSP90AA1, PTGS2, GSTP1, SOD2, MAPK3, and PCNA are all less than 0 (**Table 7**), suggesting that emodin can spontaneously bind to the amino acids of these target proteins without external assistance. Among them, the binding energies of HSP90AA1, GSTP1, SOD2, and MAPK3 are lower than those of their original ligands, while the binding energies of PTGS2 and PCNA are similar to those of their original ligands. The binding energies of emodin with these six core targets are all less than -5 kcal/mol, indicating that these core targets play important roles in the treatment of HCC by emodin. The docking results were visualized using Schrodinger software (**Figures 6**, **7**).

**Table 7:** Basic information on the molecular docking of aloe-emodin and target proteins.

Ligand	Targets	Residues	Hydrogen bond length	Binding energy (kcal/Mol)
Emodin	HSP90AA1	GLY135; TYR139LEU107; LEU103	1.99;2.22;1.81;2.09	-10.185
Emodin	PTGS2	LEU352; ARG513; TYR355	2.19;1.85;2.26	-9.733
Emodin	GSTP1	TRP28; GLN26; SER27	1.75;1.96;1.99	-7.523
Emodin	SOD2	LYS1; MET0; LYS51; SER75	1.86;2.56;1.82;2.18	-5.679
Emodin	MAPK3	LYS131; ASP184; GLN122; LYS71	2.12;1.85;1.65;1.86	-9.179
Emodin	PCNA	THR185; SER183; GLU143; LYS181	2.18; 1.96; 1.59; 2.26	-5.634
P54	HSP90AA1	ASP93; THR184	1.87;2.01	-9.398
RCX	PTGS2	TYR385; TYR355; ARG120	1.81; 1.94;1.96	-10.718
MES	GSTP1	SER27	1.85	-3.746
P04	SOD2	-	-	-3.631
6H3	MAPK3	-	-	-7.583
T2B	PCNA	-	-	-5.086

Additionally, suitable PDB format files for XRCC1, IL-6, TGFB1, and HIF1A were not found in the PDB database, hence molecular docking validation could not be performed for these targets.

#### 3.1.5 mRNA, protein expression levels and survival analysis of core targets

**Figure 8:**
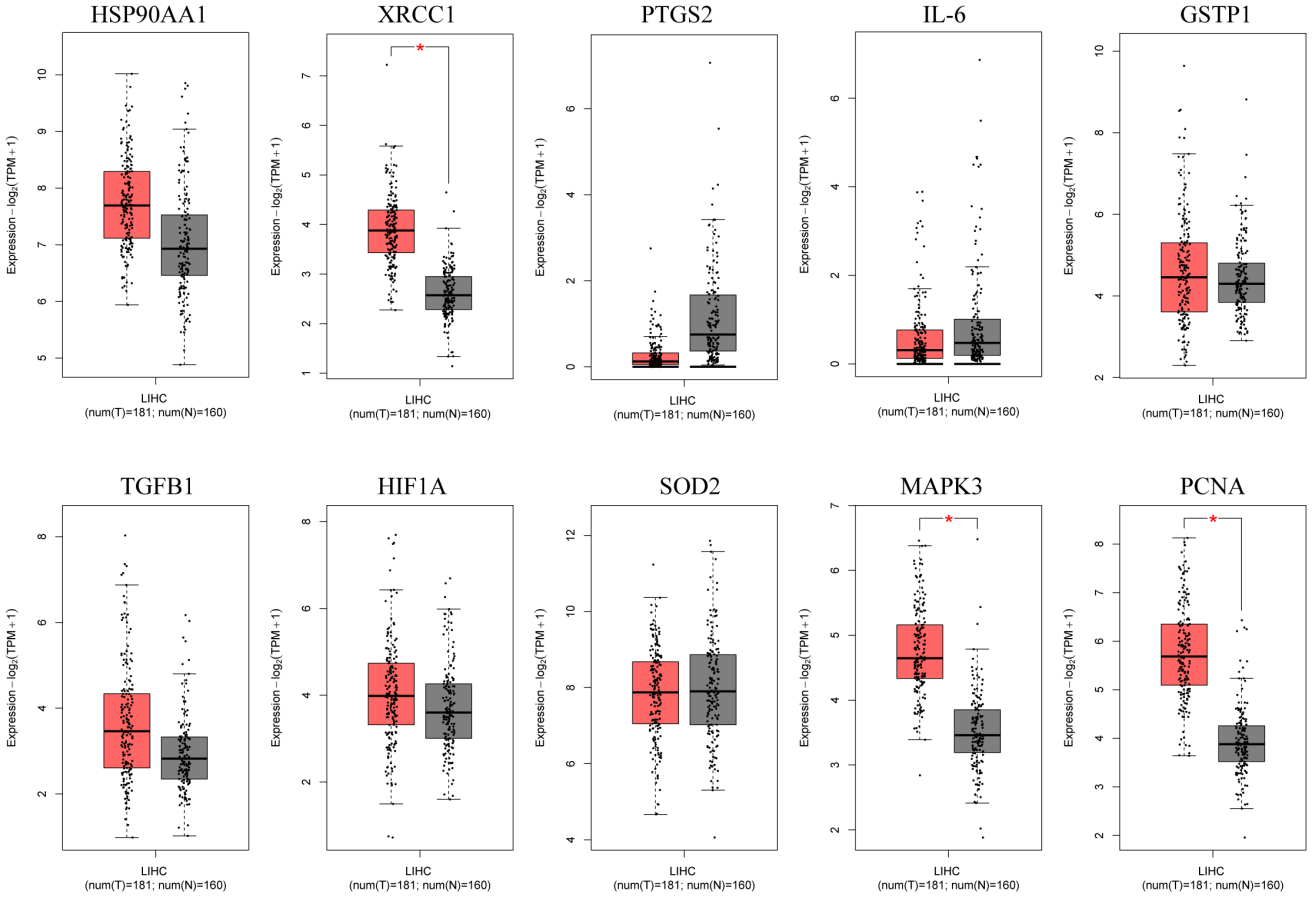
Box plots of core gene mRNA expression levels in the GEPIA database. Red represents tumor tissue, and gray represents normal tissue. The images show that compared to normal liver tissue, XRCC1, MAPK3, and PCNA are highly expressed in hepatocellular carcinoma tissue.

**Figure 9:**
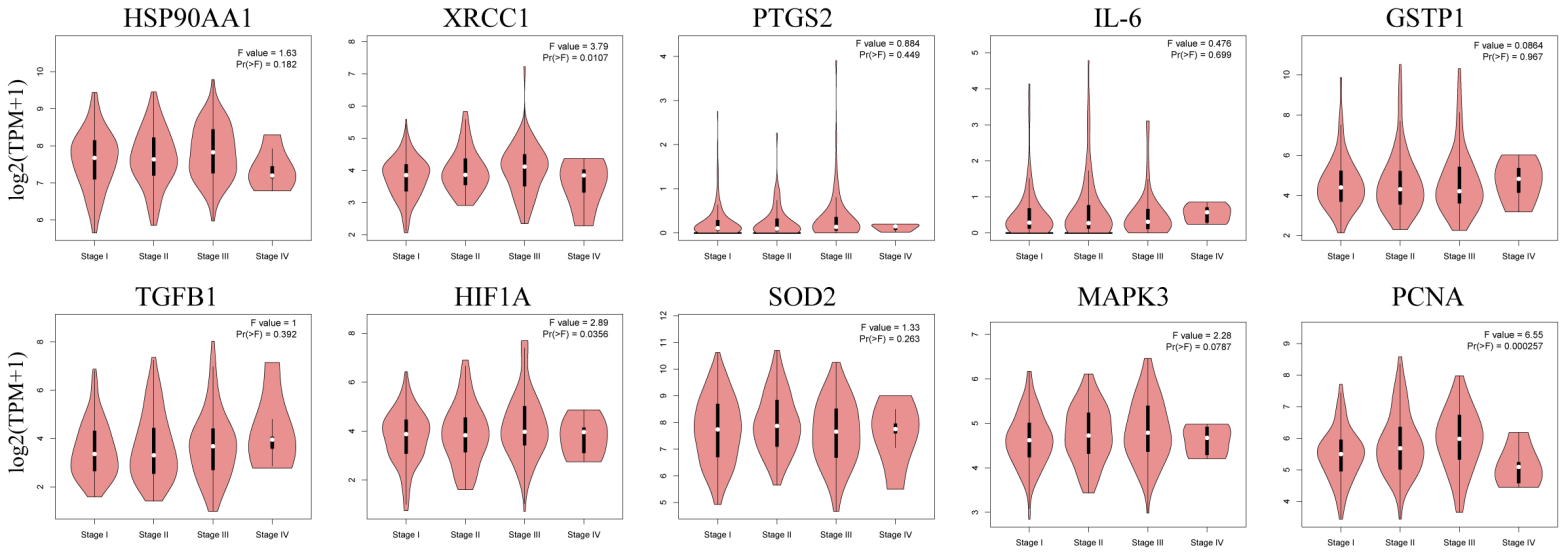
Stage plots of core gene expression and pathological stages in the GEPIA database. The expression levels of XRCC1, HIF1A, and PCNA show statistically significant differences across pathological stages (p < 0.05).

**Figure 10:**
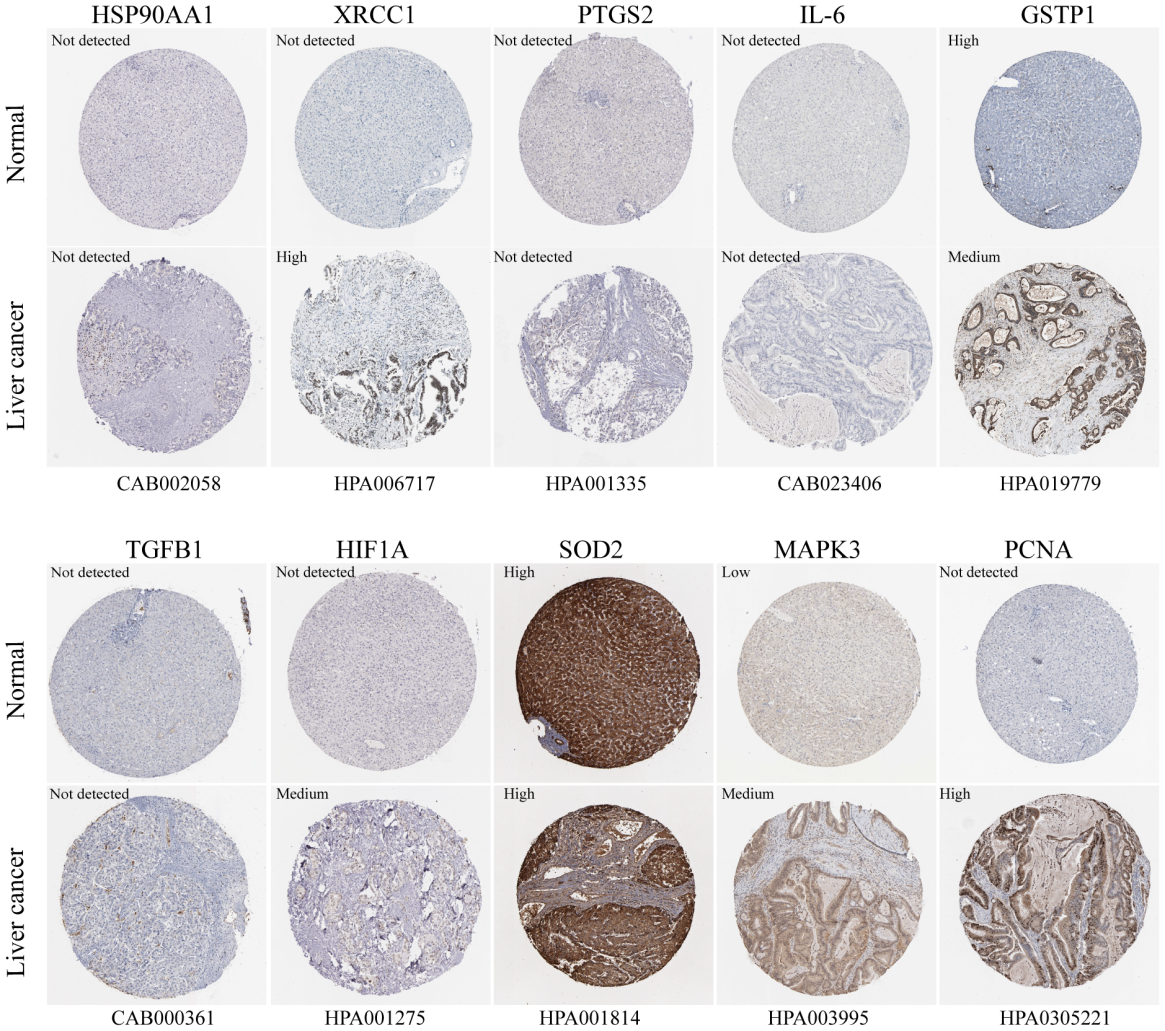
Immunohistochemistry images of core gene protein expression levels in the HPA database. The protein levels of XRCC1, HIF1A, MAPK3, and PCNA are significantly higher in HCC tissues compared to normal liver tissues, while the protein level of GSTP1 is significantly lower in HCC tissues than in normal liver tissues.

**Figure 11:**
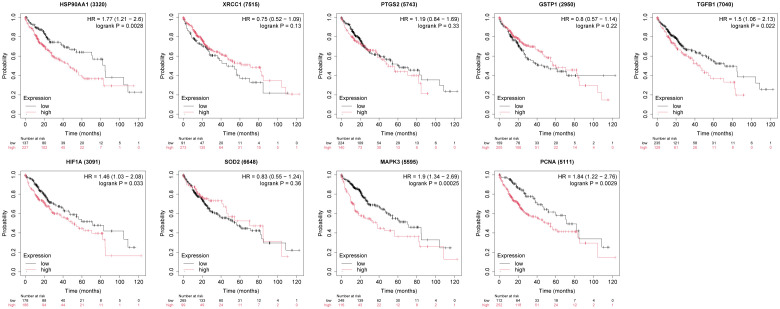
The relationship between core target expression levels and survival prognosis in hepatocellular carcinoma patients. High expression of HSP90AA1, TGFB1, HIF1A, MAPK3, and PCNA is associated with poor survival in patients.

The box plots revealed the mRNA expression of 10 core targets in normal liver tissue and HCC liver tissue. Compared to normal liver tissue, the mRNA levels of XRCC1, MAPK3, and PCNA were significantly elevated in HCC (p < 0.05) (**Figure 8**). Additionally, a correlation analysis between the mRNA expression of core targets and the progression of HCC showed significant changes in the expression of XRCC1, PCNA, and HIF1A mRNA as HCC progressed (p < 0.05) (**Figure 9**). Immunohistochemical staining images from the HPA database were used to determine the protein expression of core targets. We found that, compared to normal liver tissue, the protein expression levels of XRCC1, HIF1A, MAPK3, and PCNA were elevated, while the protein expression level of GSTP1 was reduced in HCC (**Figure 10**). Kaplan-Meier curves obtained from the Kaplan-Meier Plotter reflected the survival prognosis of HCC patients with high and low expression of core targets. Patients with high expression of HSP90AA1, TGFB1, HIF1A, MAPK3, and PCNA had a worse prognosis compared to those with low expression. This finding suggests that high expression of these five genes is an unfavorable factor for the survival of HCC patients (**Figure 11**).

### 3.2 Experimental validation

#### 3.2.1 Emodin inhibited hepatic cancer cell growth *in vitro*

**Figure 12:**
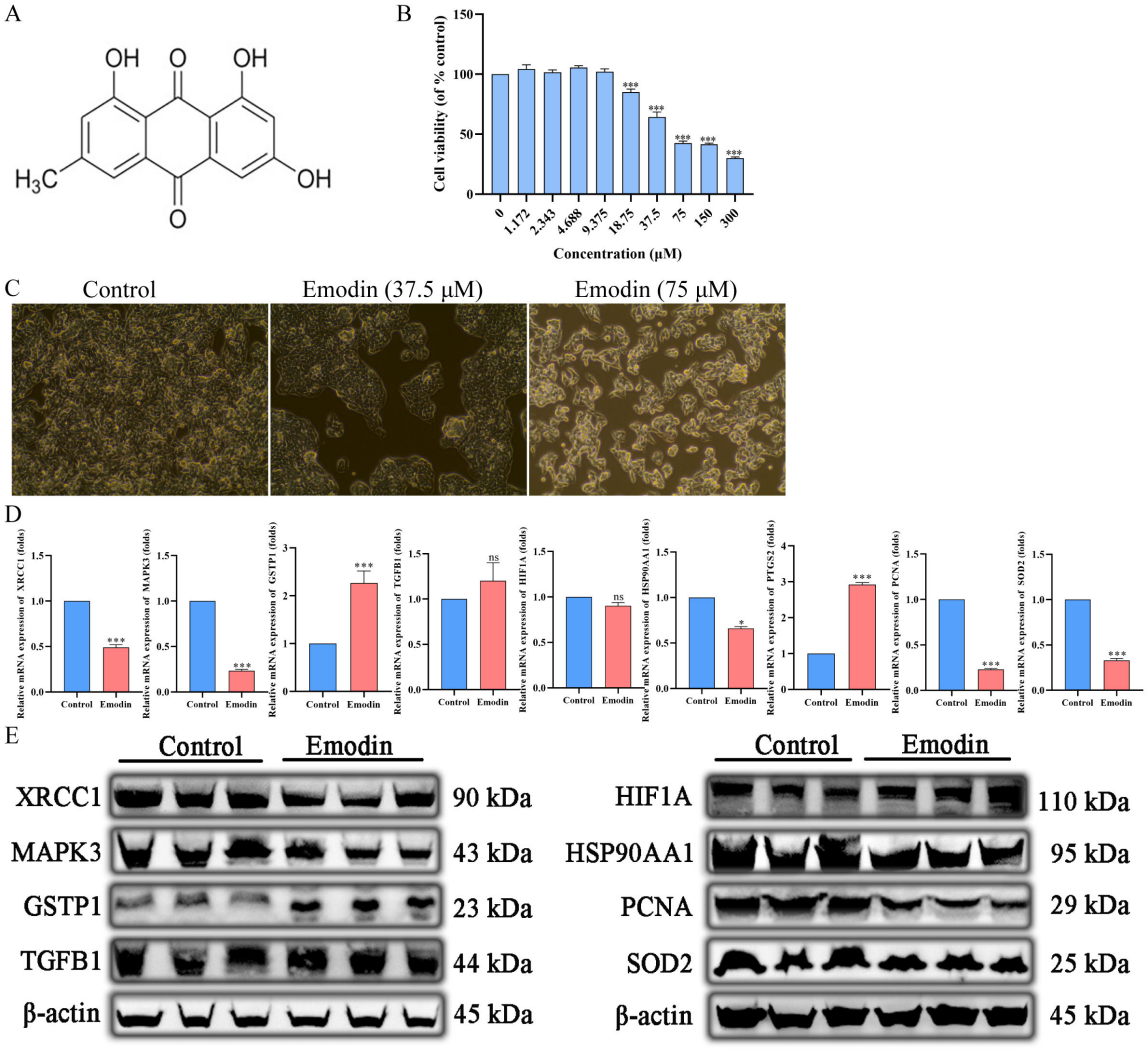
Emodin inhibits the proliferation of hepatocellular carcinoma cells. **(A)** The chemical structure of Emodin. **(B, C)** Emodin inhibits HepG2 proliferation in a concentration-dependent manner. **(D)** Emodin significantly downregulates the mRNA expression of HSP90AA1, MAPK3, XRCC1, PCNA, and SOD2 in HepG2 cells (p < 0.05), significantly upregulates the mRNA expression of PTGS2 and GSTP1 (p < 0.05), and has no significant effect on the mRNA expression of HIF1A (p > 0.05). **(E)** Emodin significantly downregulates the protein expression of HSP90AA1, MAPK3, XRCC1, PCNA, and SOD2 in HepG2 cells, significantly upregulates the protein expression of GSTP1, and has no significant effect on the protein expression of TGFB1 and HIF1A.

The chemical structure of emodin is shown in **Figure 12A**. Cell viability was assessed using the CCK-8 assay to evaluate the anticancer effects of emodin on HepG2 cells. The results showed that emodin inhibited the growth of HepG2 cells (p < 0.05), and this effect was concentration-dependent (**Figures 12B, C**). These data confirm the antiproliferative effect of emodin.

#### 3.2.2 Regulation of core target mRNA expression by emodin

The primer sequences used in this study are listed in **Table 4**. Emodin significantly downregulated the mRNA expression of HSP90AA1, MAPK3, XRCC1, PCNA, and SOD2 in HepG2 cells (p < 0.05), while it significantly upregulated the mRNA expression of PTGS2 and GSTP1 (p < 0.05). However, there was no significant effect on the mRNA expression of HIF1A (p > 0.05) (**Figure 12D**).

#### 3.2.3 Regulation of core target protein expression by emodin

The information on the antibodies used in this study is shown in **Table 7**. Emodin significantly down-regulated the expression of HSP90AA1, MAPK3, XRCC1, PCNA, and SOD2 proteins and significantly up-regulated the expression of GSTP1 proteins in HepG2 cells, whereas it had no significant effect on the expression of TGFB1 and HIF1A proteins (**Figure 12E**).

## 4 Discussion

Hepatocellular carcinoma (HCC) is the most common type of primary liver cancer, with an increasing incidence worldwide and a poor prognosis. Hepatitis B virus (HBV) infection is the most common cause of HCC globally. Although existing treatments (such as surgery, radiotherapy, and chemotherapy) have extended patients’ survival to some extent, their efficacy is limited and they often have significant side effects. Therefore, exploring new therapeutic approaches is of paramount importance. Emodin, a natural anthraquinone compound, has been shown in numerous studies in recent years to have significant effects in inhibiting HCC cell proliferation, inducing apoptosis, and suppressing tumor angiogenesis. Our research aims to investigate the molecular mechanisms of emodin’s anti-HCC effects using network pharmacology and molecular docking studies.

In this study, we first identified 43 genes associated with emodin and HBV-HCC. These genes may serve as key targets for emodin in the treatment of HBV-HCC. Enrichment analysis of these 43 genes suggests that emodin may treat HBV-HCC by positively regulating cytokine production, cell development, and cell migration. Previous research partially supports these findings. For example, studies by Xiao Hongbin et al. demonstrated that emodin can activate the NFκB signaling pathway and induce IL-6 upregulation (Zhang et al., 2019). Additionally, research by Ni Jian et al. showed that emodin can significantly regulate the expression of cell cycle-related proteins (such as CDK2, CDK4, CDK6) in hepatocellular carcinoma cells and normal hepatocytes (Lin et al., 2017; Li et al., 2022). Through KEGG enrichment analysis, we found that the main pathways involving these 43 genes include cancer pathways, human cytomegalovirus infection pathways, tuberculosis pathways, IgA production intestinal immune network pathways, gastric cancer pathways, prion disease pathways, and prostate cancer pathways. Using protein-protein interaction (PPI) network analysis, we identified 10 core targets: HSP90AA1, XRCC1, PTGS2, IL-6, GSTP1, TGFB1, HIF1A, SOD2, MAPK3, and PCNA. PPI analysis indicates that these proteins, with higher interaction intensities, are more likely to be targeted for emodin in the treatment of HBV-HCC. Subsequently, we used molecular docking to further verify the interaction strength between emodin and these 10 proteins. The results showed that the binding energies of HSP90AA1, PTGS2, GSTP1, SOD2, MAPK3, and PCNA were all less than -5 kcal/mol, with binding energies smaller or similar to those of their original ligands. This indicates that these six proteins are more likely to be the targets of emodin in the treatment of HBV-HCC.

HSP90 interacts with and supports various proteins that promote cancer, making it crucial for malignant transformation and tumor progression. Consequently, HSP90 inhibitors show promising prospects in the treatment of various cancers (Zuehlke et al., 2015; Xie et al., 2023). Our study demonstrates that emodin can significantly inhibit the expression of HSP90AA1. Furthermore, studies have shown that emodin is a novel small-molecule agonist that induces programmed necrosis in prostate cancer cells through the mitochondrial fission HSP90-MLKL-PGAM pathway (Zhou et al., 2023). XRCC1 is a DNA repair scaffold that supports base excision repair and single-strand break repair and is also involved in other repair pathways, playing a central role in the BER pathway (Whitehouse et al., 2001). Research on gliomas has found that emodin can induce apoptosis within 48 hours, but resistance develops after 48 hours, potentially due to the high expression of XRCC1 in glioma cells. However, there is currently no research on the specific effects of emodin on XRCC1 (Kuo et al., 2009). Although studies have shown that XRCC1 polymorphism does not affect the prognosis of HCC, the mRNA expression level of the XRCC1 gene is significantly correlated with patient prognosis (Zhao et al., 2019; Mei et al., 2020). Our study is the first to report that emodin can significantly inhibit the expression of XRCC1 mRNA and protein in HepG2 cells. PTGS2 also known as COX2, is a prognostic marker for renal cancer and plays multiple roles in cancer cell resistance to chemotherapy and radiotherapy. Emodin can inhibit HCC growth by downregulating COX2 and activating PINK1/Parkin-mediated apoptosis (Chen et al., 2019; Hashemi Goradel et al., 2019; Hu et al., 2021). This finding is not consistent with our study. The specific reasons for this discrepancy require further investigation. GSTP1 is an isoenzyme of glutathione S-transferase that protects cells from cytotoxic and carcinogenic substances. Overexpression of GSTP1 can inhibit HCC cell proliferation by blocking the cell cycle, but it has also been shown to inhibit apoptosis (Tao et al., 2016; Liu et al., 2018). Emodin can effectively inhibit the expression of GSTP1, thereby reducing the incidence of glutathione-related resistance in human leukemia (Duvoix et al., 2004). However, our results show that emodin significantly upregulates the expression of GSTP1, which may be related to the concentration of emodin used. TGF-β1 encodes transforming growth factor β1 (TGF-β1), a potent cytokine that regulates various cellular processes, including proliferation, differentiation, wound healing, and immune responses. TGF-β1 can activate the canonical SMAD pathway or generate non-canonical signaling cascades (Schmierer and Hill, 2007). The peculiarity of TGF-β1 lies in its context dependency; in healthy epithelial tissue and early stages of tumorigenesis, TGF-β1 negatively regulates the proliferation and growth of precancerous epithelial cells, thereby inhibiting tumors. In contrast, in advanced cancer, tumor cells can reconnect the TGF-β1 pathway to avoid apoptosis and suppress immune responses, thus promoting tumor progression (Tufegdzic Vidakovic et al., 2015). Emodin can reduce glucose-induced TGF-β1 expression (Chan et al., 2003). HIF1A promotes the growth and metastasis of HCC by participating in VEGF-mediated control of cell proliferation, angiogenesis, and invasion (Forsythe et al., 1996; Ma et al., 2022). Emodin can alleviate hypoxia-induced embryotoxicity by upregulating HIF1A (Yon et al., 2011). However, this study did not find that Emodin has a significant regulatory effect on TGF-β1 and HIF1A. SOD2, found only in the mitochondrial matrix, is associated with mtDNA and is thought to prevent the oxidation of mtDNA and mtDNA polymerase. The mRNA expression of SOD2 is reduced in HBV-HCC patients, and studies have shown that SOD2 is closely related to HCC prognosis (Wang et al., 2016). However, this was not observed in our research. However, our study confirmed that emodin significantly downregulates the mRNA and protein expression of SOD2 in HepG2 cells. MAPK3, also known as extracellular signal-regulated kinase 1 (ERK1), is located downstream of the Raf/MEK/ERK pathway and regulates cell proliferation, differentiation, and survival, amplifying signals during tumor invasion and metastasis (Yang et al., 2019). Emodin significantly downregulates the gene expression of MAPK3. PCNA is an important target for DNA replication and repair, and its high expression is closely related to poor prognosis, which is consistent with our research (Ma et al., 2016). Emodin can promote liver regeneration by upregulating PCNA (Meng et al., 2005). Emodin may treat hepatocellular carcinoma by inhibiting the expression of PCNA.

Before this, studies on emodin’s regulatory effects on the genes mentioned above in HCC were virtually nonexistent. This study is the first to systematically investigate emodin’s regulatory impact on these core targets, suggesting potential directions for future research.

## 5 Conclusion

This study explored the molecular mechanisms of Emodin in the treatment of HBV-related hepatocellular carcinoma (HBV-HCC) based on network pharmacology and molecular docking. The results indicate that this process involves multiple signaling pathways. Emodin may exert its therapeutic effects on HBV-related HCC by downregulating the expression of XRCC1, MAPK3, PCNA, HSP90AA1, and SOD2, and upregulating the expression of PTGS2 and GSTP1. This study provides further theoretical support for the clinical application and mechanistic exploration of Emodin in the treatment of HBV-HCC.

## Data Availability

The raw data supporting the conclusions of this article will be made available by the authors, without undue reservation.
